# Puppet resting behavior in the Tibetan antelope (*Pantholops hodgsonii*)

**DOI:** 10.1371/journal.pone.0204379

**Published:** 2018-12-27

**Authors:** Yunchao Luo, Lin Wang, Le Yang, Ming Tan, Yiqian Wu, Yuhang Li, Zhongqiu Li

**Affiliations:** 1 School of Life Sciences, Nanjing University, Nanjing, China; 2 Tibet Plateau Institute of Biology, Lhasa, China; Sichuan University, CHINA

## Abstract

Rest contributes a large part of animals’ daily life, and animals usually rest in two ways, standing or in recumbence. Small or medium sized ungulates bed to rest in most cases, and standing rest is very rare and hardly seen. Here we described a standing rest behavior of Tibetan antelopes (*Pantholops hodgsonii*) living on the Tibet Plateau which has not been reported before. We named the standing rest behavior Puppet behavior, since the antelope stand still for a certain time. Of the 304 individuals observed, 48.3% (98/203) of adult and sub-adult males expressed the Puppet behavior, whereas only 6.3% (6/96) of females did, indicating an obvious sexual difference. Puppet behavior occurred more frequently at noon and in the afternoon on sunny and cloudy days, meaning that daytime and weather were both influential factors. Puppet behavior was usually accompanied with rumination and sometimes ended with leg-shaking. Our results suggest that Puppet behavior may be an adaptive form of rest, which may serve a thermoregulatory and anti-predation function, and may be simpler and safer than recumbent rest.

## Introduction

Animals usually behave in a relatively fixed manner, and these common behaviors can be classified into several categories including feeding, resting, moving, alerting, grooming, etc. Animals need rest or sleep to perform a number of physiological functions such as saving energy, thermoregulation, and maintenance of their immune system [1, 2]. Generally, animals need to sleep or take rest for 2 to more than 20 hours a day to recover from the exhaustion due to daily activities [3–6]. Even during the daytime, animals spend a large amount of their time to rest [3].

Rest is a perilous condition of reduced activity, little consciousness, and greatly reduced responsiveness during which predation is a risk [7, 8]. Therefore, animals need to adopt a proper way and time to rest, in order to recover physiologically and avoid their predators. Animals take rest either by standing or bedding (in recumbence) [9]. Standing rest highlights the animal for observation by predators [7]. Thus to adopt standing rest probably depends upon the anti-predator ability of the animals or more specifically their body size. Small or medium sized cloven-hoofed animals tend to bed to rest. For example, Przewalski’s gazelle (*Procapra przewalskii*) with a body size of about 26 kg, spend 38% of their day time to rest in recumbence [10]. When they stand, they are usually dealing with feeding or alert, and we had never seen them rest while standing. This is also true in other small or medium sized ungulates including Tibetan gazelle (*P*. *picticaudata*) [11], Asiatic ibex (*Capra sibirica*) [12, 13], and goitred gazelle [14]. However, standing rest seems common in large body-sized ungulates like the Asiatic wild ass (*Equus hemionus*) which spend nearly one third of their rest time standing [14]. Similarly, elephants have an average daily total sleep (standing or in recumbence) time of 2 h due to the large body size, and only exhibit recumbent sleep every third or fourth day [15–17].

The Tibetan antelope is a flagship species to Qinghai-Tibet Plateau. The total population underwent a severe decline in the 1980s and early 1990s as a result of commercial poaching for the valuable underfur [18]. Rigorous protection has been enforced since then, and recent estimates were made at about 100,000 ~ 150,000 [19, 20]. Tibetan antelope has been classified as Near Threatened by IUCN since 2016 and is a Category I (Endangered in China) National Protected Wild Animal Species in China since 1989. Tibetan antelope breeds from December to January and the lambing season is from June to July. They are dimorphic; adult male with an average body weight of 39kg, is larger than female with an average of 26 kg [20]. The resident status of Tibetan antelope can be divided into two types, the migratory population and the resident population. Our focal population in Shenzha of Tibet is resident and does not migrate. The population might be up to 10, 000.

The most significant mammalian predator in this area is the wolf (*Canis lupus*), which is relatively common; the snow leopard (*Panthera uncia*), the lynx (*Felis lynx*) and the brown bear (*Ursus arctos*), which are much rarer. Tibetan fox (*Vulpes ferrilata*), are also common and may prey upon lambs of the antelopes. Large raptors including upland buzzard (*Buteo hemillasius*), cinereous vulture (*Aegypius monachus*) and lammergeier (*Gypaetus barbatus*) are common and are frequent scavengers of dead ungulates.

**Fig 1:**
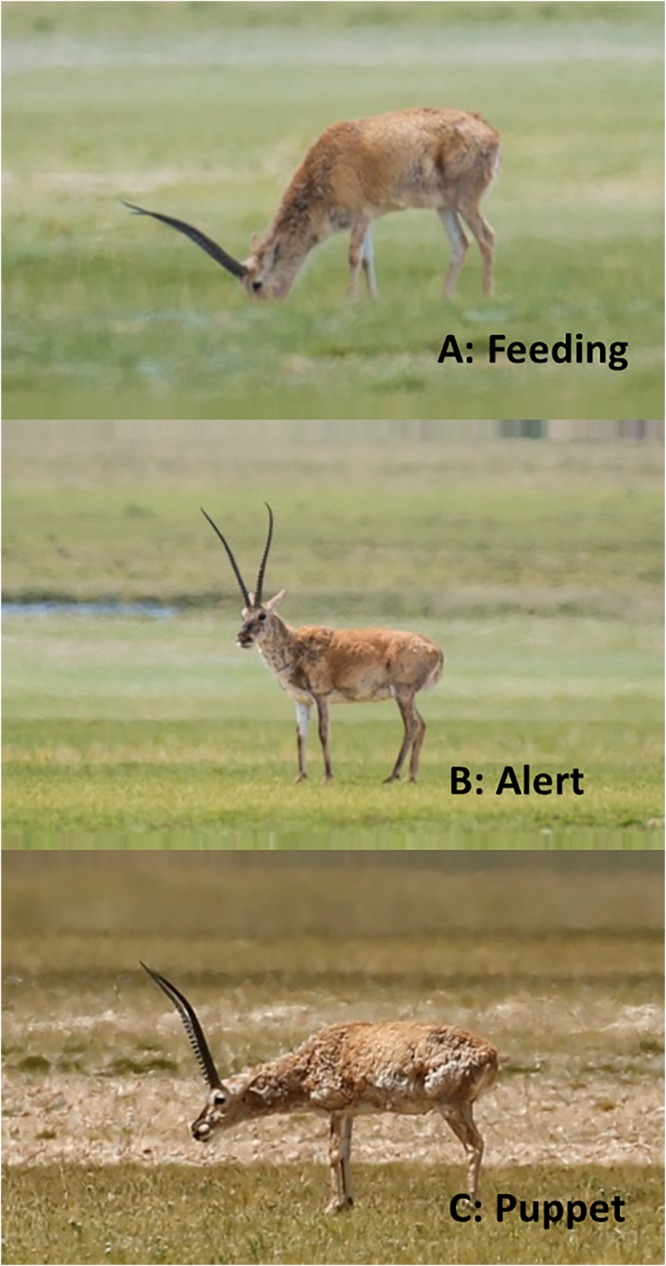
Photos of feeding (A), vigilance (B) and Puppet behavior (C) of Tibetan antelopes.

We started our field behavioral study on Tibetan antelopes in 2016 and found that Tibetan antelopes especially males sometimes kept standing for several min (S1 Movie and Fig 1). During this time, they didn’t feed, didn’t move, and just kept their body motionless, like a puppet. Here we collected two years data to explore if the occurrence of Puppet behavior is related to the day time, weather and sex-age.

## Materials and methods

### Study areas

This study was conducted in Shenzha County (30°02′39″~32°19′33″ N, 87°45′30″~89°47′49″ E) located in the central part of Tibet Autonomous Region, China. Elevations range from 4,530m to 6,448m, with an average of 4700m. Local climate is characterized by extreme cold and long winters, strong winds and high levels of solar radiation. Mean annual temperature is 0.4°C. Annual precipitation is about 330 mm and most rain falls between June and September. Alpine meadow is the main vegetation type and no shrubs appear in this area.

### Behavioral sampling

Daytime observations were carried out from sunrise to sunset in the summer (July & August of 2016, June & July of 2017) in Shenzha. We defined a group as a herd of antelopes with no more than 50 m separating any two group members. Observations were carried out from the roadside using binoculars (8X42) or a telescope (20~60X63). We walked or drove regular routes to find targets for video recording.

For most groups, we focused observations on one or two individuals. We collected a few more observations from some large groups with more than tens or a few hundred individuals, but we observed individuals from different parts of the group to avoid resampling. It was not practical or feasible to mark individuals or to recognize individuals through particular features, and thus there was a possibility that individuals were observed or recorded more than once. However, since the population was more than 10 000, the possibility of resampling in a same day was rather small.

At the beginning of each observation period, we recorded the date, time of day, location, weather, group type, and group size. Only three group types (single-male groups, single-female groups and mother-lamb groups) could be found during summer. Observed individuals were classified into four age categories: adult male, sub-adult male, female and lamb. It was not possible to distinguish adult females from sub-adult females, and thus the two age classes were combined.

Behavioral events were video recorded. Observations lasted 30 min unless we lost sight of the individual. Puppet behavior was defined as standing still, head up or down, and without any other apparent acts.

### Ethical approval

This is an observational experiment and all observations were made at a distance of more than 200 m. All the experiment procedures in this study were approved by the Chinese Wildlife Management Authority.

### Statistical Analysis

We observed 304 individuals over 3968 min with an average (± SE) observation length of 13.1 ± 0.2 min. We established a logistic regression model to evaluate the effects of time (morning: before local time 11:00; noon: local time 11:00–14:00; afternoon: after local time 14:00, weather (sunny: sunshine without cloud; cloudy: sunshine with cloud; overcast: thick cloud; rainy), sex-age (adult male, sub-adult male, female, and lamb), and group size (1–400 with a median of 7) on the occurrence of Puppet behavior and the occurrence of recumbent rest. The link function for the logistic models can be expressed as log-odds = β_0_+β_1_X_time_+β_2_X_weather_+β_3_X_sex-age_+β_4_X_groupsize_. For individuals that expressed at least one occurrence of Puppet behavior (n = 104), we established a general linear model to see if the duration of Puppet behavior was affected by time, weather, sex-age, and group size. All these analysis were done using SPSS 19.0, values were shown with Mean ± SE, and significant levels were set at 0.05.

## Results

The Puppet behavior was observed 344 times in 104 individuals (S1 Dataset).

**Fig 2:**
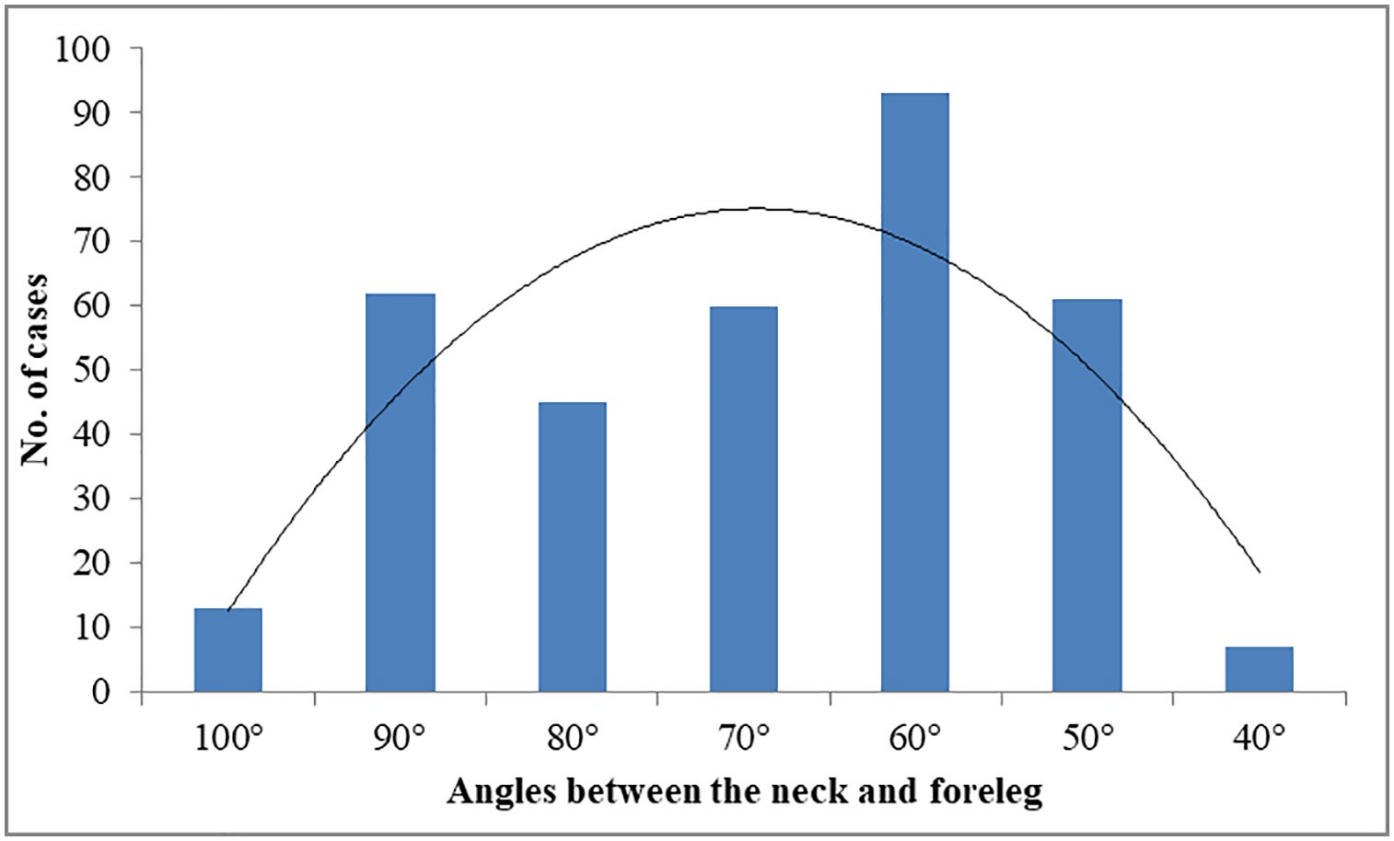
Posture expression of Puppet behavior of Tibetan antelopes.

Puppet behavior was defined as standing still, without any acts of their whole body for a certain time (ranged from 2 to 842 s, with a median of 57 s). We measured the angle between their neck and foreleg, and found that the angle was usually between 40° and 100° (Figs 1 and 2). Comparably, when the antelopes were feeding, the angle was usually less than 40°; and when they were in vigilance, the angle was always more than 120°, with head up and scanning around. Most (63.9%, 92/144) of the clearly observed Puppet behaviors were found to be accompanied by oral rumination (S2 Movie). Nearly a third of the antelopes ended the Puppet behavior by shaking their hind legs (102/344, 29.7%).

**Fig 3:**
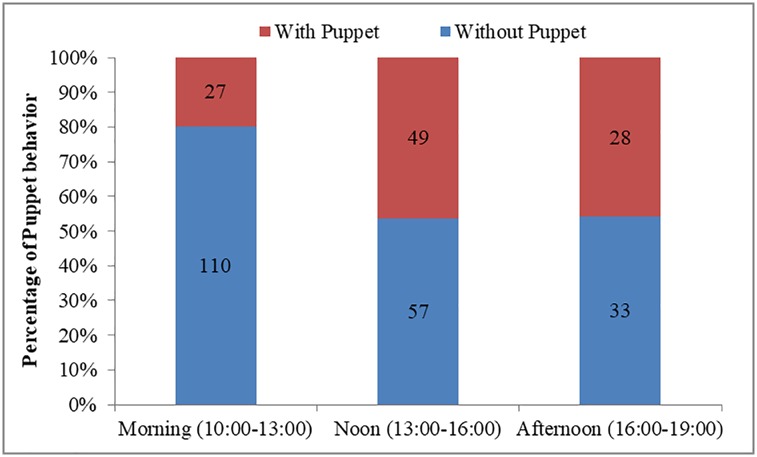
Effect of day time on the occurrence of Puppet behavior of Tibetan antelopes. Note: all were in Beijing Time; local time is two hours later than Beijing Time.

**Fig 4:**
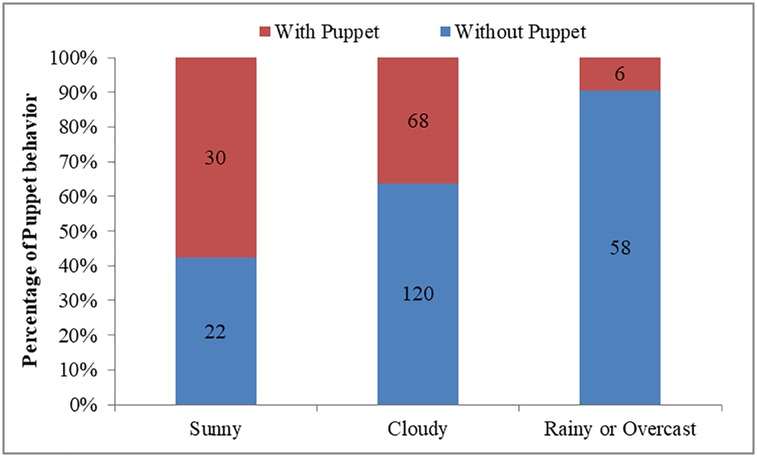
Effect of weather on the occurrence of Puppet behavior of Tibetan antelopes.

**Fig 5:**
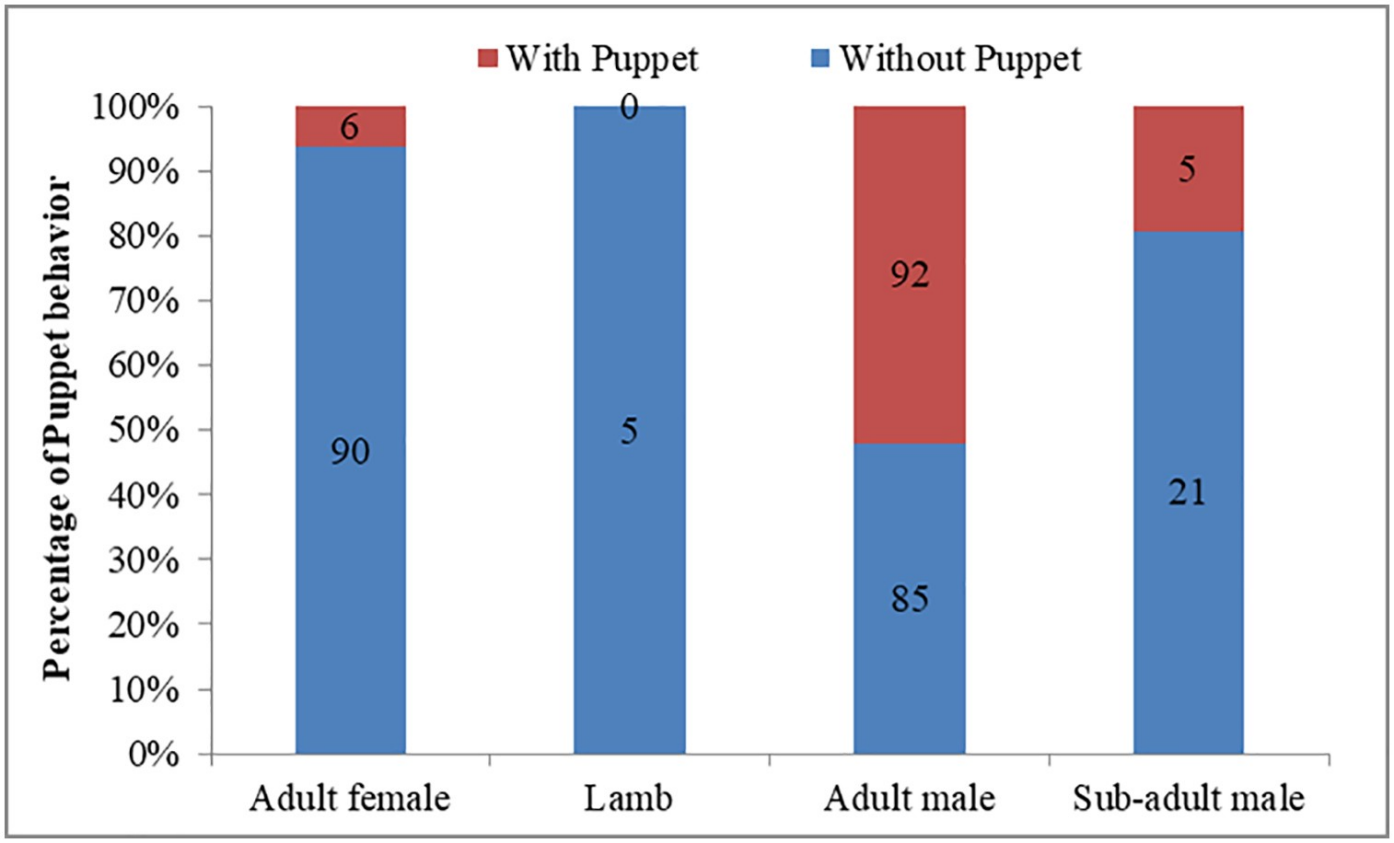
Effect of sex-age on the occurrence of Puppet behavior of Tibetan antelopes.

The occurrence of Puppet behavior was affected by time (Wald = 8.114, df = 2, P = 0.017), weather (Wald = 22.121, df = 2, P<0.001), and sex-age (Wald = 23.984, df = 3, P<0.001), but not group size (Wald = 1.279, df = 1, P = 0.258). Compared to the afternoon (28/61, 45.9%; Fig 3), Puppet behavior occurred less in the morning (27/137, 19.7%; β = -1.261±0.461, Wald = 7.500, df = 1, P = 0.006). Compared to overcast or rainy days (6/64, 9.4%; Fig 4), Puppet behaviors was observed more often on sunny days (30/52, 57.7%; β = 2.404±0.545, Wald = 19.486, df = 1, P<0.001) or cloudy days (68/188, 36.2%; β = 2.119±0.487, Wald = 18.949, df = 1, P<0.001). Adult females (6/96, 6.25%; Fig 5) expressed much more Puppet behaviors than adult males (93/177, 52.5%; B = 2.503±0.572, Wald = 19.133, df = 1, P<0.001) and similar rates as sub-adult males (5/26, 19.2%; β = 1.103±0.759, Wald = 2.115, df = 1, P = 0.146). Lambs did not express any Puppet behavior (0/5, 0%).

The occurrence of recumbent rest was independent of time (Wald = 1.726, df = 2, P = 0.422), sex-age (Wald = 0.250, df = 2, P = 0.969), group size (Wald = 1.974, df = 1, P = 0.160), but marginally influenced by weather (Wald = 5.962, df = 2, P = 0.051)., The antelopes bedded more on sunny days than rainy days (β = 0.911±0.447, Wald = 4.159, df = 1, P = 0.041).

The duration of Puppet behavior in females lasted 42.7±41.5 s, compared to adult males (91.5±14.1 s) or sub-adult males (100.3±43.3 s), however, the difference was not significant (F_2, 96_ = 0.736, P = 0.482). Similarly, there was no significant effect of weather (F_2, 96_ = 0.429, P = 0.652), day time (F_2, 96_ = 2.462, P = 0.091) and group size (F_1, 96_ = 0.035, P = 0.851).

## Discussion

We found a very interesting behavior of Tibetan antelopes, which was named Puppet behavior since they didn’t feed, didn’t move, and just kept their body motionless, like a puppet (S1 Movie and Fig 1). However, this Puppet behavior seemed very different from vigilance, since their head also kept motionless and was usually under shoulder, and thus probably cannot detect a whole of their surroundings. To our knowledge, there is not any report on this kind of standing behavior in Tibetan antelopes, or other median or small sized ungulates. We considered that the Puppet behavior serves a primary function of rest. Our results indicate that the Puppet behavior of Tibetan antelope occurred more frequently at noon and in the afternoon, on sunny and cloudy days, and in males, indicating that all the three factors (day time, weather, and sex-age) shaped the expression of Puppet behavior.

Animals rest and sleep in their own way, bedding or standing, and the resting position may be dependent on their body size. Generally large body-sized ungulates rest in both standing and recumbent ways, and the standing rest can contribute to an even larger part than recumbent rest, e.g, Przewalski’s horses (*E*. *przewalskii*) spent 15.7% of a whole day and up to 25% of their diurnal time in standing rest [21]; Giraffes spent about 20% of their time in recumbent rest, but standing was also a very important way of rest, in mothers, standing rest even contributed to 1/3 of their night time from 4:00 to 7:00 [9]. However, standing rest seems very rare in median or small sized ungulates, and recumbence is probably the main or the only way to rest, as we found in other Tibetan ungulates [10].

The frequent occurrence of standing rest by Tibetan antelopes, especially by males, seems difficult to interpret. We proposed two possible explanations—energy saving and anti-predation, both closely related to body size. With the increase of body size, rest appears to progress from standing to recumbence and then back to standing, this could be costly in terms of both time and energy. Just like small animals run with crouched postures, whereas larger species run more upright, due to the difference of scaling of bone and muscle geometry [22]. This extremely slow progress is very risky and may lead to a fatality in the case of an immediate attack. Standing rest for a large body-sized animals exposes them as obvious targets for the predators. However, with larger body sizes; the risk of predation may be lower compared to smaller animals. Standing may enable large animals to have a better visual field allowing identification of predators earlier and timely reactions.

In Tibetan antelopes, body size principle may also be operating. Larger male Tibetan antelope exhibited standing rest for much more frequently than smaller females. Body size, as well as its associated ability of anti-predation, probably becomes the determinant why males preferred standing rest.

Animals with larger body size would probably have evolved an adaptive body structure to maintain balance and save energy while standing to rest [23]. Just like horses, the skeleton is helpful to support and balance themselves when they are resting with standing state [23]. For Tibetan antelopes, we consider that the standing state might be a relaxed way to rest because the angle between their neck and foreleg was around 70°, not like vigilance behavior with an angle of more than 90°or feeding behavior with an angle of less than 40°, both of which need an intense coordination of both skeletal and muscular systems. This posture of Tibetan antelope is in congruence with the standing rest of flamingos by single leg [24], which needs more mechanical analysis.

Both time of day and weather can influence Puppet behavior, but they were not the determinants since the behavior also occurred in the early morning and on rainy or overcast days. It is possibly that the lower occurrence of Puppet behavior in the early morning may be due to the previous night sleep period and they need more time to feed. The rest starts at noon and occurs frequently in the afternoon. The Puppet behavior occurs more frequently when the weather is sunny or cloudy, and it may serve a function of thermoregulation. The elevation in this area is very high and the average temperature is relative low compared with other areas [25]. However, the sun radiation is rather strong and the real-time temperature reached 20°C during sunny days. While standing, the exposed surface area of the antelopes is larger resulting in more body contact with the air which can facilitate heat dissipation. This is true in other ungulates, such as Asiatic wild ass [26], Przewalski’s horse [27, 28], African wild ass (*E*. *africanus*) [29] and Grevy’s zebra (*E*. *grevyi*) [30].

The duration of the standing rest ranged from 1–2 min, and most of the time (63.9%; 92/144) standing rest was accompanied by oral rumination. Rumination usually occurs while at rest, both standing or in recumbence [31]. Actually EEG recordings have shown that rumination is not exclusive with rest [9]. Puppet rest at a standing posture may be relaxing for the antelope to ruminate their food and recover their body, as well as to be ready to escape should an attack occur [7, 32]. However, this assumption requires confirmation.

Leg-shaking behavior was also found sometimes when the antelopes ended the Puppet behavior. This leg-shaking behavior is similar to the relaxed rolling behavior of horses, which also occurs after sleeping [33, 34]. The beluga whale (*Delphinapterus leucas*) expresses muscle jerks occurred more frequently at the end of rest episodes [35].

## Conclusion

We report a strange but interesting Puppet behavior in Tibetan antelopes. This kind of standing rest behavior can be found more frequently in large sized ungulates but occurred in medium sized Tibetan antelopes. Several factors including sex-age, day time and weather can influence the expression of Puppet behavior. Further studies should focus on the mechanical analysis on the standing architecture to explore how the antelopes balance themselves while rest.

## Supporting information

S1 Dataset Raw data. (XLSX)

S1 Movie Display Puppet behavior together. (AVI)

S2 Movie Puppet behavior with rumination. (AVI)
